# Development and validation of a machine learning model for sperm DNA fragmentation rate in infertile men: a multicenter retrospective study

**DOI:** 10.3389/fendo.2026.1838500

**Published:** 2026-06-22

**Authors:** Ke Wang, Jinxia Zheng, Xuanxuan Ge, Jie Bai, Mengmeng Ma, Ningxin Qin, Xin Huang, Hui Jiang, You Zhang

**Affiliations:** 1 Center of Reproductive Medicine, Shanghai Key Laboratory of Maternal Fetal Medicine, Shanghai Institute of Maternal-Fetal Medicine and Gynecologic Oncology, Shanghai First Maternity and Infant Hospital, School of Medicine, Tongji University, Shanghai, China; 2 Taian Central Hospital, The Affiliated Taian City Central Hospital of Qingdao University, Taian, China; 3 School of Medicine, Tongji University, Shanghai, China; 4 Center of Reproductive Medicine, Xinhua Hospital Affiliated Shanghai Jiao Tong University, School of Medicine, Shanghai, China; 5 Information Center, Shanghai Key Laboratory of Maternal Fetal Medicine, Shanghai Institute of Maternal-Fetal Medicine and Gynecologic Oncology, Shanghai First Maternity and Infant Hospital, School of Medicine, Tongji University, Shanghai, China

**Keywords:** infertile men, machine learning, predictive model, risk factors, sperm DNA fragmentation rate

## Abstract

**Background:**

The sperm DNA fragmentation index (DFI) provides important reference for evaluating male fertility and assisted reproductive outcomes, and its degree of damage is influenced by multiple factors. Our study aims to develop a machine learning-based predictive model for identifying high sperm DFI in infertile men using clinical and semen parameters.

**Methods:**

We retrospectively collected data on infertile male patients from two centers in Shanghai, China from March 2023 to March 2024. We used data from one center as the training cohort to construct the model and data from another center for external validation. The semen data of the included subjects is combined with clinical features as training features for machine learning. We have developed and validated the effectiveness of six models: Decision Tree (DT), Random Forest (RF), eXtreme Gradient Boosting (XGBoost), Support Vector Machine (SVM), Logistic Regression (LR), and Naive Bayes Classifier (NB). We comprehensively evaluated the performance of machine learning models with different features using ROC curves, accuracy, and other relevant indicators. The SHapley Additive exPlanations (SHAP) diagram was used to illustrate the importance of variables in the model, Lasso regression is used to screen for core features. Finally, a convenient and practical DFI quality early prediction platform was constructed based on core features.

**Findings:**

1037 patients from one center were included in the development cohort, while 290 patients from another center were included in the external validation queue. The RF model performed the best in predicting the quality of DFI in infertile male patients, with a 10−fold cross−validation AUC of 0.979 (0.972−0.986) in the development cohort, and an AUC of 0.945 (95% CI: 0.916−0.975) in the external validation cohort. Finally, the core factors obtained through screening were included in the model, including progressive motility sperm rate, sperm concentration, sperm viability, daily exercise time, smoking status, alcohol consumption, stress level, and insomnia symptoms were incorporated to generate a publicly accessible online platform (https://4sjajo-0-0.shinyapps.io/dfi-prediction-v4/).

**Interpretation:**

The RF model using semen parameters and lifestyle factors shows good discrimination for predicting DFI abnormality in infertile men. However, the model exhibits notable miscalibration in external validation (calibration slope 2.196, intercept -0.196), indicating systematic overestimation of risk and insufficient dispersion of predictions. Therefore, in its current form, the model and its associated online calculator should be considered investigational. Prospective validation and recalibration in independent populations are required before any clinical application. Its impact on patient−important reproductive outcomes (e.g., live birth) remains unknown.

## 1 Background

Declining fertility has become a major global issue and severe challenge. In high-income countries, the incidence of infertility ranges from 3.5% to 16.7%, while in low-income countries, it ranges from 6.9% to 9.3% (1). By 2020, the infertility rate in China had reached as high as 17.6% (2). Among these cases, male-specific factors account for approximately 30%, and combined male and female factors account for about 20% (3). The number of male infertility patients continues to rise, making rapid and accurate assessment of sperm quality an important challenge in the field of reproductive medicine. The World Health Organization (WHO) Laboratory Manual for the Examination and Processing of Human Semen (6th edition) recommends incorporating the sperm DNA fragmentation index (DFI) into sperm quality testing (4). Sperm DNA integrity may be more predictive of male fertility potential than conventional measurements of sperm morphology and motility, particularly in predicting successful pregnancy rates following assisted reproductive technology (ART) treatments for males with unexplained infertility (5). However, sperm DFI is highly sensitive to oxidative stress damage and influenced by multiple factors (6). Therefore, identifying risk factors associated with sperm DFI and constructing a predictive model for sperm DFI quality are particularly critical for infertile men undergoing ART treatment.

Traditional sperm analysis methods exhibit significant limitations in classification efficiency, objectivity, and cost-effectiveness, failing to meet clinical demands. The introduction of artificial intelligence (AI) technology offers innovative solutions for this field. Machine learning, a branch of AI, represents the scientific approach that enables computer systems to learn from data and improve their performance. Currently, machine learning has been applied to predict semen quality parameters including semen volume, sperm concentration, and total sperm count (7). Recent studies have integrated quantitative phase imaging (QPI) with convolutional neural network models, allowing real-time measurement of dynamic sperm DNA fragmentation index (DFI) levels without staining requirements. This advancement preserves sperm functionality for subsequent intracytoplasmic sperm injection (ICSI), overcoming the limitation of sperm non-viability caused by acridine orange staining (8). These developments further demonstrate the significant effectiveness of AI applications in male infertility research. However, existing methodologies remain operationally complex, clinically inaccessible, and lack incorporation of objective lifestyle-related indicators inherently present during the conception preparation phase in infertile males.

Therefore, this study aims to analyze multidimensional risk factors affecting DNA fragmentation index (DFI) quality in infertile males through easily accessible clinical data. Multiple machine learning algorithms will be employed to construct predictive models for DFI quality in infertile men. Each model will be systematically evaluated to identify the optimal model, which will then undergo validation of its clinical applicability. This research endeavors to provide a more scientific basis for clinical decision−making, although the ultimate impact on reproductive success requires further study.

## 2 Study object

### 2.1 Study design and cohorts

This multicenter retrospective study was conducted using data from two reproductive medicine centers in Shanghai, China:

#### 2.1.1 Development cohort

Center 1 – Reproductive Medicine Center of Tongji University Affiliated Obstetrics and Gynecology Hospital. Data were collected from March 2023 to December 2023.

#### 2.1.2 External validation cohort

Center 2 – Reproductive Medicine Center of Xinhua Hospital Affiliated to Shanghai Jiao Tong University School of Medicine. Data were collected from January 2024 to March 2024.

The development cohort was used for model training and internal validation, while the external validation cohort was held out entirely and used only for final model evaluation. All patients in both centers met the same inclusion and exclusion criteria (detailed below). Semen testing procedures followed the WHO Laboratory Manual (6th edition) (4) with identical reagents and equipment across centers, and laboratory personnel received standardized training from the same manufacturer.

### 2.2 Study participants

#### 2.2.1 Inclusion criteria

① Met the diagnostic criteria for infertile males according to the World Health Organization Laboratory Manual for the Examination and Processing of Human Semen (6th edition) (4), and received ICSI treatment; ② Had normal male reproductive system and physical examination results, with no medical history affecting sperm quality; ③ Had not undergone treatments or used medications that could affect sperm quality prior to semen analysis; ④ Voluntarily participated in this study.

#### 2.2.2 Exclusion criteria

① Chromosomal or genetic abnormalities detected through preimplantation genetic testing; ② Patients using donor sperm, surgical sperm retrieval, or frozen sperm for assisted reproduction; ③ Patients with severe chronic diseases, tumors, or other comorbidities; ④ Patients who refused to participate in the questionnaire survey.

### 2.3 Ethical approval

This study strictly followed indication criteria and complied with all relevant laws, regulations, and ethical principles. The guidelines outlined in the Declaration of Helsinki were adhered to. All participants provided written informed consent, and the study protocol was approved by the Ethics Committee of Tongji University Affiliated Obstetrics and Gynecology Hospital (Ethics Approval No.: KS2313) and by the Ethics Committee of Xinhua Hospital Affiliated to Shanghai Jiao Tong University School of Medicine (Approval No.: XHEC-NSFC-2024-231), and this study complies with the Helsinki Declaration.

### 2.4 Methods

#### 2.4.1 Sample size calculation method

T The sample size was justified using an events-per-variable (EPV) approach, as discussed in the framework proposed by Riley et al. (9) for developing clinical prediction models. A total of 30 candidate predictor parameters were initially considered for model development, including demographic characteristics, lifestyle factors, psychological scales, serum testosterone, and routine semen parameters (as listed in **Tables 1**, **2**). Based on the observed data, the anticipated proportion of patients with abnormal DFI (DFI ≥ 25%) in the development cohort was 33.2%. Adopting a conservative EPV criterion of at least 10, the minimum required number of events was calculated as:

**Table 1:** Distribution of general clinical characteristics in infertile male patients (n=1327).

Item	Group	Cases	Proportion (%)
Age (year)	20∼25	6	0.45
	26∼35	773	58.25
	36∼45	476	35.87
	>45	72	5.43
BMI (kg/m2)	≤18.5	21	1.58
	18.6∼23.9	398	29.99
	24.0~27.9	604	45.52
	≥28.0	304	22.91
Educational level	High school and below	443	33.38
	College/Undergraduate	643	48.46
	Master’s degree or above	241	18.16
Cola	Yes	300	22.61
	No	1027	77.39
Tea	Yes	754	56.82
No	573	43.18
Coffee	Yes	399	30.07
	No	928	69.93
Smoking (cigarettes/d)	None	544	40.99
	0~10	237	17.86
	11~20	266	20.05
	>20	280	21.10
Alcohol drinking (mL/d)	None	665	50.11
	0~50	227	17.11
	51~100	224	16.88
	>100	211	15.90
High heat and high radiation working environment	Yes	53	3.99
	No	1274	96.01
Hot Spring	Yes	75	5.65
	No	1252	94.35
Daily sleep time (h/d)	<7	578	43.56
	7~9	684	51.54
	>9	65	4.90
Daily Exercise Time (h/d)	Almost none	346	26.07
	0~0.5	475	35.80
	0.6~1	327	24.64
	>1	179	13.49
Anxiety	None	778	58.63
	Mild	317	23.89
	Moderate	160	12.06
	Severe	72	5.42
Depression	None	773	58.25
	Mild	386	29.09
	Moderate	135	10.17
	Severe	33	2.49
Stress	None	332	25.02
	Moderate pressure	523	39.41
	High pressure	428	32.25
	Heavy pressure	44	3.32
Insomnia	None	320	24.11
	Suspected Insomnia	525	39.56
	Insomnia	482	36.32

**Table 2:** Distribution of sperm quality parameters in infertile male patients (n=1327).

Item	$\bar{x}$± s	Classification	Min	Max
Testosterone	5.54 ± 1.98		2.01	12.94
Progressive Motility Sperm Rate (%)	23.27 ± 5.98	/	3.22	31.9
Non-Progressive Motility Sperm Rate (%)	17.38 ± 6.24	/	5.36	47.8
Immotile Sperm Rate (%)	59.44 ± 9.03	/	24.31	79.80
Head deformity rate (%)	79.38 ± 3.91	/	66.00	90.00
Mixed malformation rate (%)	18.24 ± 3.82	/	6.00	30.00
Normal sperm morphology rate (%)	2.83 ± 0.95	/	0.00	6.00
Sperm Viability rate (%)	48.62 ± 7.99	/	10.00	68.00
Sperm Concentration (106/mL)	12.01 ± 6.76	/	2.00	64.40
Semen volume (ml)	3.52 ± 1.51	/	0.30	11.50
Semen PH	7.29 ± 0.20	/	7.00	8.00
Leukocytospermia	6.54 ± 1.73	/	3.21	16.71
Mycoplasma	/	Negative	1147	86.43%
	/	Positive	180	13.56%
Chlamydia	/	Negative	1149	86.59%
	/	Positive	178	13.41%

Required events = 30 (parameters) × 10 = 300.

The corresponding minimum total sample size would be 300/0.332≈904. Our development cohort (n = 1037) contained 344 events, yielding an EPV of 344/30 = 11.47, which exceeds the recommended minimum of 10. Therefore, the sample size is adequate to support the complexity of the planned prediction model and to minimize the risk of overfitting. Furthermore, the use of 10−fold cross−validation and penalized regression (LASSO) provides additional safeguards against overfitting. We note that this is a conservative EPV-based justification rather than a full parametric sample size calculation (e.g., using the pmsampsize package), because the latter would require assumptions about the anticipated model R² that are difficult to specify *a priori*. The EPV≥10 criterion is widely accepted for providing stable estimates of predictor effects in logistic regression–based prediction models.

#### 2.4.2 Survey instruments

① General demographic data: A self-designed questionnaire based on the “Chinese Expert Consensus on Male Fertility Assessment” (10) included 12 items: age, body mass index (BMI), educational level, smoking history, alcohol consumption history, habitual cola consumption (>500 mL/day), habitual strong tea consumption (>500 mL/day), habitual coffee consumption (>500 mL/day), average daily exercise duration, average daily sleep duration, exposure to high-risk occupational environments (high temperature, radiation/radioactive exposure), and sauna habits (>1 session per week). ② Self-Rating Anxiety Scale (SAS) and Self-Rating Depression Scale (SDS) (11): Based on symptoms experienced in the past week, total raw scores were calculated by summing all 20 items, with standardized scores = raw score ×1.25. According to Chinese norms, SAS standardized scores of 50–59 indicated mild anxiety, 60–69 moderate anxiety, and ≥70 severe anxiety; SDS standardized scores of 53–62 indicated mild depression, 63–72 moderate depression, and >72 severe depression. ③ Athens Insomnia Scale (AIS) (12): This self-rating scale assessed patient conditions over the past month. Total scores ranged from 0–24 across 8 items: 0–3 indicated no sleep disorder, 4–6 suggested suspected insomnia, and total scores >6 defined insomnia. ④ Chinese Perceived Stress Scale (CPSS) (11): Developed by Cohen et al. in 1983 and revised by Yang et al. in 2003, this scale demonstrated good structural validity with Cronbach’s α=0.780. Comprising 2 dimensions and 14 items, total scores of 10–14 indicate no pressure, 15–28 points indicate moderate pressure, 29–42 points indicate high pressure, and 43–56 points indicate heavy pressure. ⑤ Serum total testosterone levels: Measured using chemiluminescence immunoassay after venous blood collection (3 mL fasting sample) and serum separation. ⑥ Semen quality analysis: Routine semen analysis followed World Health Organization laboratory manual standards (6th edition) (4). After 3–4 days of sexual abstinence, semen samples were collected via masturbation into sterile containers. Post-liquefaction, 10μL aliquots were loaded onto counting chambers for sperm concentration and motility assessments. Normal sperm morphology rates were determined through hematoxylin-eosin (HE) staining, while sperm survival rates used eosin-aniline black staining. DFI uses sperm chromatin structure assay (SCSA) method for monitoring.

#### 2.4.3 Survey methods

Prior to initiating the investigation, reproductive medicine specialists and psychotherapists provided training on relevant knowledge to the participating healthcare professionals. After obtaining informed consent from the study subjects, researchers administered questionnaires for patients to complete independently on the day of medical record establishment, ensuring adequate time allocation and a private environment. Questionnaires were collected immediately upon verification of completeness without missing items. Relevant laboratory data were retrieved through electronic medical records. To ensure data accuracy and integrity, all collected information was entered into Excel spreadsheets by two independent personnel for subsequent organization and analysis. All participants signed written informed consent forms, with questionnaire responses linked to laboratory results via anonymous ID codes to maintain confidentiality.

This study strictly implements the quality control process, and the laboratory testing of the two centers is completely in accordance with World Health Organization Laboratory Manual for the Examination and Processing of Human Semen (6th edition) Conduct semen testing using the same manufacturer and batch of semen testing reagents, and provide training on testing techniques through technical personnel from the same manufacturer; In addition, it is strictly required that the study subjects abstain from sexual activity for 3–4 days to avoid excessive or insufficient abstinence that may affect the research results.

Based on Evenson’s research (13), the SCSA method commonly uses 25% as the upper threshold and has been cited or recommended by multiple guidelines and expert consensus. Therefore, this study considers DFI≥25% as the observation group and DFI<25% as the control group.

#### 2.4.4 Statistical methods

Data were analyzed using R4.2.2 and Python 3.6.9 software. According to data distribution characteristics, normally distributed metric data are presented as mean ± standard deviation ( $\overset{–}{x}$ ± s); non-normally distributed metric data are expressed as median [interquartile range (IQR)]; categorical variables are summarized as frequency (percentage) [n (%)].

##### 2.4.4.1 Data preprocessing and splitting

After excluding samples with missing key clinical or laboratory data, the dataset from Center 1 (n = 1037) served as the development cohort. The dataset from Center 2 (n = 290) served as the external validation cohort and was not accessed during any phase of model development or internal validation.

##### 2.4.4.2 Handling of missing data, outliers, and data cleaning

**Figure 1:**
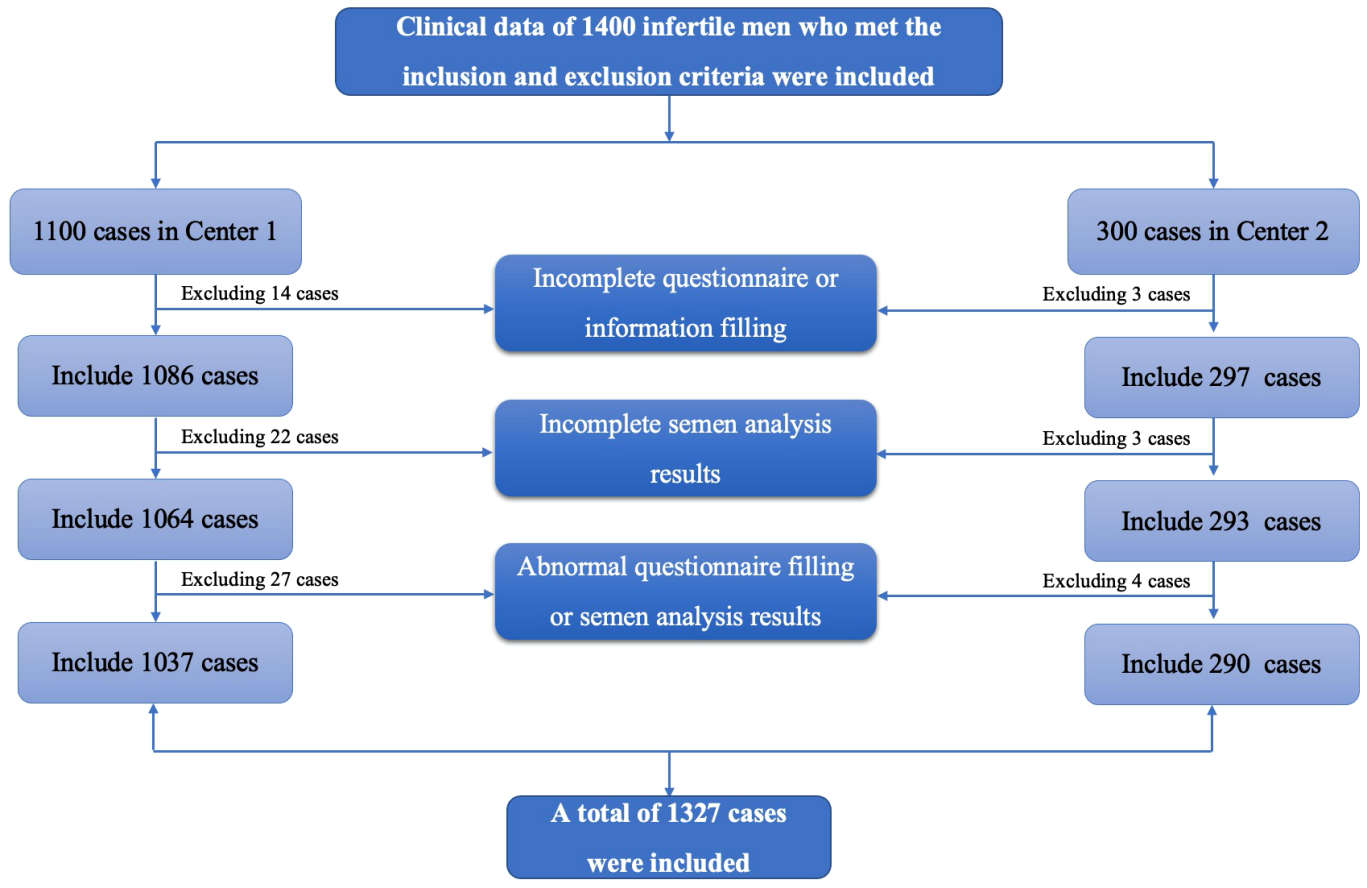
Process flow chart for subject inclusion.

A total of 1,400 infertile male patients were initially enrolled from both centers. After data cleaning, 1,327 (94.8%) patients were retained for analysis. The inclusion flowchart is shown in **Figure 1**. The reasons for exclusion of the remaining 73 patients (5.2% of the initial sample) were as follows:

① Incomplete questionnaire or information filling (17cases, Center 1: 14, Center 2: 3): identity information not filled in (7cases, Center 1: 6, Center 2: 1), BMI not filled in (5cases, Center 1:5), psychological questionnaire not filled in (4cases, Center 1: 3, Center 2: 1), and lifestyle assessment not filled in (1cases, Center 1: 1).

② Incomplete semen analysis results (25cases, Center 1: 22, Center 2:3): No semen examination was conducted at the investigation center or valid semen examination data was not obtained.

③ Abnormal questionnaire filling or semen analysis results (31cases, Center 1: 27, Center 2: 4): selecting the same option for all items in validated scales (24 cases, Center 1: 21, Center 2: 3), using drugs that affect sperm quality before semen examination (4 cases, Center 1:3, Center 2:1), and non-compliance with the required 3-4 days of abstinence before semen examination (3 cases, Center 1: 3).

Among the 73 excluded patients, based on available partial data, 20 (27.40%) would have been classified as DFI≥25% and 53 (72.60%) as DFI<25%, suggesting no substantial differential exclusion by outcome. In the final sample, complete−case analysis was performed without imputation.

##### 2.4.4.3 Internal validation using nested cross−validation

To obtain unbiased estimates of model performance while avoiding data leakage, we employed a nested 10−fold cross−validation (CV) procedure that incorporates feature selection, hyperparameter tuning, and model evaluation within each outer loop. The development cohort (n = 1037) was randomly partitioned into 10 outer folds. For each outer fold, the following steps were performed on the remaining 9 folds (inner training set):

###### 2.4.4.3.1 Feature selection

LASSO logistic regression with 10−fold inner cross−validation (on the current inner training set) was applied to select predictors. The optimal penalty parameter λ was chosen as lambda.1se. This step identified the set of non−zero predictors, which typically included the eight variables consistently selected across folds: Progressive Motility Sperm Rate, Sperm Concentration, Sperm Viability, Daily Exercise Time, Smoking, Drinking, Stress, and Insomnia.

###### 2.4.4.3.2 Hyperparameter tuning

Using only the selected predictors from step 1, each of the six machine learning algorithms (DT, RF, XGBoost, SVM, LR, NB) was tuned via a second-level 10-fold inner CV (again within the current inner training set). The hyperparameter combination maximizing the mean CV-AUC was selected for each algorithm.

###### 2.4.4.3.3 Model fitting and evaluation

For each algorithm, the final model with the selected predictors and optimal hyperparameters was trained on the entire inner training set (9 outer folds) and then evaluated on the held−out outer fold (the 10th fold).

This nested CV procedure was repeated 10 times, each time with a different outer fold serving as the validation set. The predicted probabilities from all 10 outer validation folds were aggregated to compute the overall performance metrics (AUC, accuracy, sensitivity, specificity, F1 score, Brier score, calibration intercept, calibration slope) for each algorithm. Furthermore, Decision curve analysis (DCA) was performed to evaluate the clinical utility of each model under a single predefined clinical decision scenario: offering a confirmatory sperm DFI test (which is costly or resource−intensive) to patients whose predicted probability of DFI abnormality exceeded a given threshold probability. We report standardized net benefit, defined as the absolute net benefit divided by the maximum possible net benefit at that threshold (i.e., the net benefit achievable if all patients with the outcome were correctly identified). Standardized net benefit ranges from 0 to 1 and facilitates comparison across thresholds and models. The net benefit of using the model to guide this testing decision was compared against two default strategies: (1) “treat none” – offer DFI testing to no patients, and (2) “treat all” – offer DFI testing to all patients. No other clinical decisions (e.g., treatment selection, ART strategy, or lifestyle modification) are implied by this analysis. Confidence intervals were obtained by bootstrapping (2,000 resamples) from the aggregated predictions. Calibration curves and decision curve analysis were also based on these cross-validated predictions. Notably, the set of eight predictors was consistently selected across most outer folds; for consistency and interpretability, the final models reported in this study were refitted on the entire development cohort using these eight predictors and the optimal hyperparameters determined from the nested CV procedure. This approach provides a nearly unbiased estimate of expected model performance on new data.

###### 2.4.4.3.4 External validation

The final models (trained on the full development cohort) were applied directly to the external validation cohort (Center 2, n=290) without any modification. The same performance metrics were calculated on this independent dataset.

###### 2.4.4.3.5 Interpretability and calculator

SHapley Additive exPlanations (SHAP) were computed for the RF model using the development cohort to quantify feature contributions. An interactive web−based risk calculator was developed using the Shiny framework (R package version 1.7.4) and deployed on shinyapps.io. The calculator uses the eight LASSO−selected predictors and the final RF model to generate individual risk predictions. It is freely accessible at https://4sjajo-0-0.shinyapps.io/dfi-prediction-v4/ (for research purposes only). The following coding scheme was used in all machine learning models (including the final model), SHAP analysis, external validation, and the online risk calculator:

① Continuous variables (entered as numeric values): Progressive Motility Sperm Rate (%), Sperm Concentration (×10^6^/mL), Sperm Viability (%).

② Ordinal categorical variables (entered as integer scores preserving order) (see **Table 3**):

**Table 3:** Ordered classification variable assignment table.

Predictor variable	Assignment status
Daily Exercise Time	0 = “almost none”, 1 = “0–0.5 h/d”, 2 = “0.6–1 h/d”, 3 = “>1 h/d”
Smoking	0 = “none”, 1 = “0–10 cigarettes/d”, 2 = “11–20 cigarettes/d”, 3 = “>20 cigarettes/d”
Alcohol Drinking	0 = “none”, 1 = “0–50 mL/d”, 2 = “51–100 mL/d”, 3 = “>100 mL/d”
Stress	0 = “none”, 1 = “moderate pressure”, 2 = “high pressure”, 3 = “heavy pressure”
Insomnia	0 = “none”, 1 = “suspected insomnia”, 2 = “insomnia”

For ordinal categorical variables (Daily Exercise Time, Smoking, Alcohol Drinking, Stress, Insomnia), integer scores (0,1,2,3) were assigned to preserve the natural order across categories. This coding assumes an ordered relationship (e.g., higher stress scores indicate greater perceived stress) but does not assume equal intervals between scores. Dummy−variable encoding was not used for these variables, in order to maintain model simplicity and interpretability.

This coding scheme was applied consistently across all stages: LASSO feature selection (with ordinal variables treated as numeric), hyperparameter tuning, nested cross-validation, final model refitting, SHAP analysis, external validation, and deployment of the web-based calculator. The online calculator (https://4sjajo-0-0.shinyapps.io/dfi-prediction-v4/) uses identical categorical options and continuous value ranges, ensuring that the risk prediction for any input corresponds exactly to the output of the final RF model.

### 2.5 Handling of missing or invalid inputs in the deployed model

The online risk calculator (https://4sjajo-0-0.shinyapps.io/dfi-prediction-v4/) requires complete input for all eight predictors. No imputation is performed. If any predictor value is missing or outside the pre−specified valid range (e.g., progressive motility <0% or >100%, sperm concentration <0, categorical options outside the defined integer codes), the calculator returns an error message and does not produce a risk prediction. Range checks are implemented for all continuous variables. This conservative “complete−case” approach aligns with the development phase (complete−case analysis) and avoids extrapolation or undocumented imputation.

## 3 Results

### 3.1 Basic characteristics of infertile men

1327 valid samples were obtained. Among these, the development cohort (Center 1) consisted of 1037 cases, of whom 344 (33.17%) had DFI≥25%. The external validation cohort (Center 2) consisted of 290 cases, with 95 (32.76%) having DFI≥25%. Detailed clinical characteristics are presented in **Tables 1**, **2**.

### 3.2 Lasso regression analysis of DFI in infertile men

**Figure 2:**
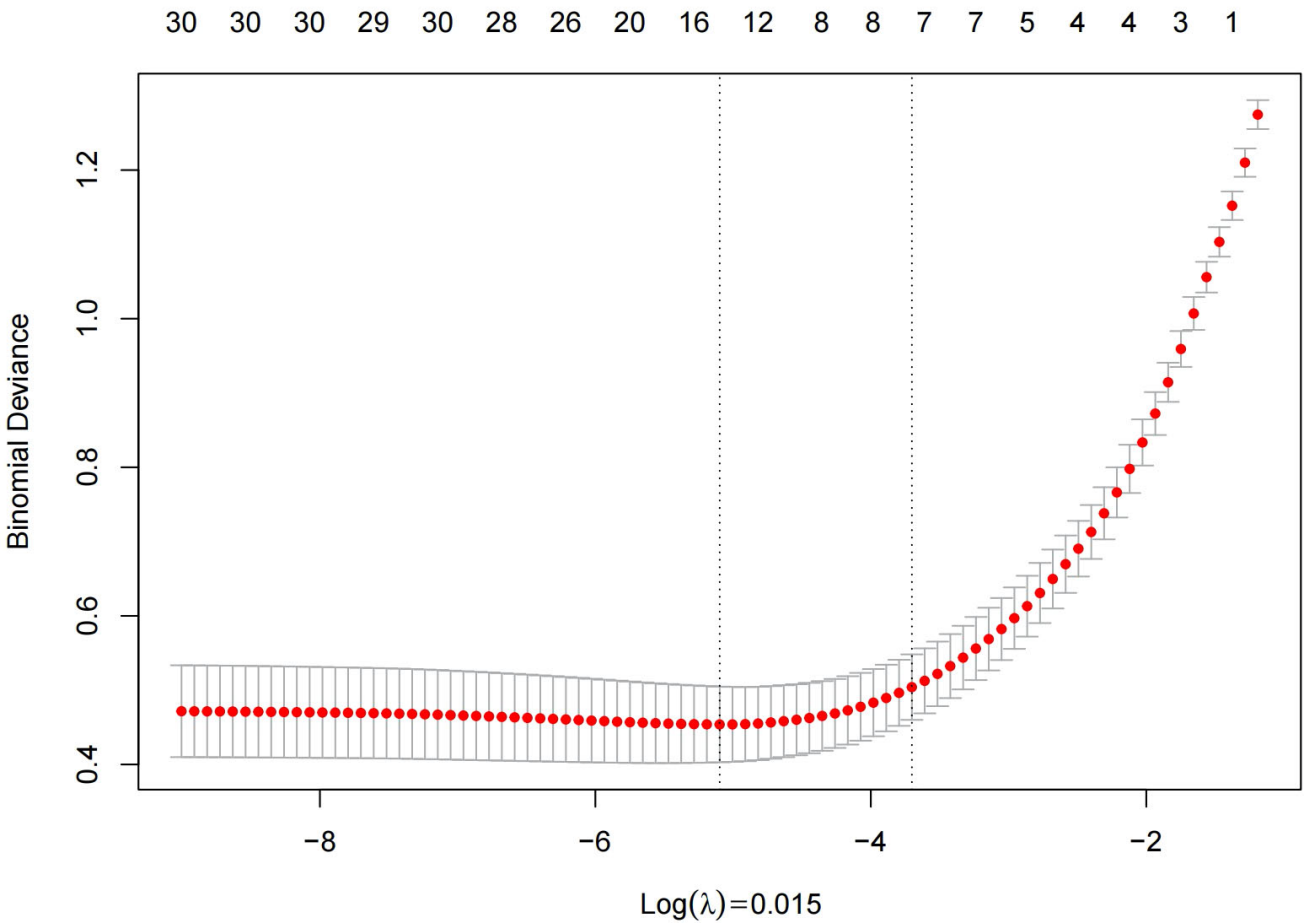
Hazard factor selection based on LASSO regression.

**Figure 3:**
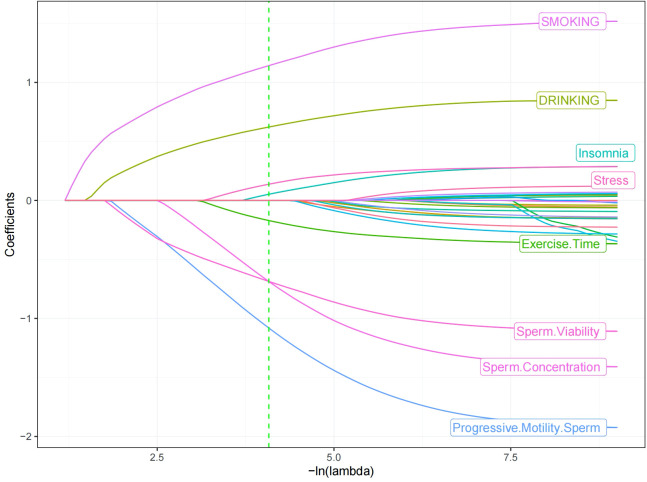
Coefficient distribution.

To visualize the coefficients and confirm the directionality of the selected predictors, a final LASSO logistic regression model was fitted on the entire development cohort (n = 1037) using 10−fold cross−validation to select the penalty parameter (lambda.1se). This analysis, which is independent of the nested cross−validation procedure described in Methods, confirmed the same eight predictors that were consistently selected across the outer folds of the nested CV: Progressive Motility Sperm Rate, Sperm Concentration, Sperm Viability, Daily Exercise Time, Smoking, Drinking, Stress, and Insomnia. The coefficients from this final LASSO fit are shown in **Figures 2**, **3**.

### 3.3 Establishment and evaluation of the predictive model

**Figure 4:**
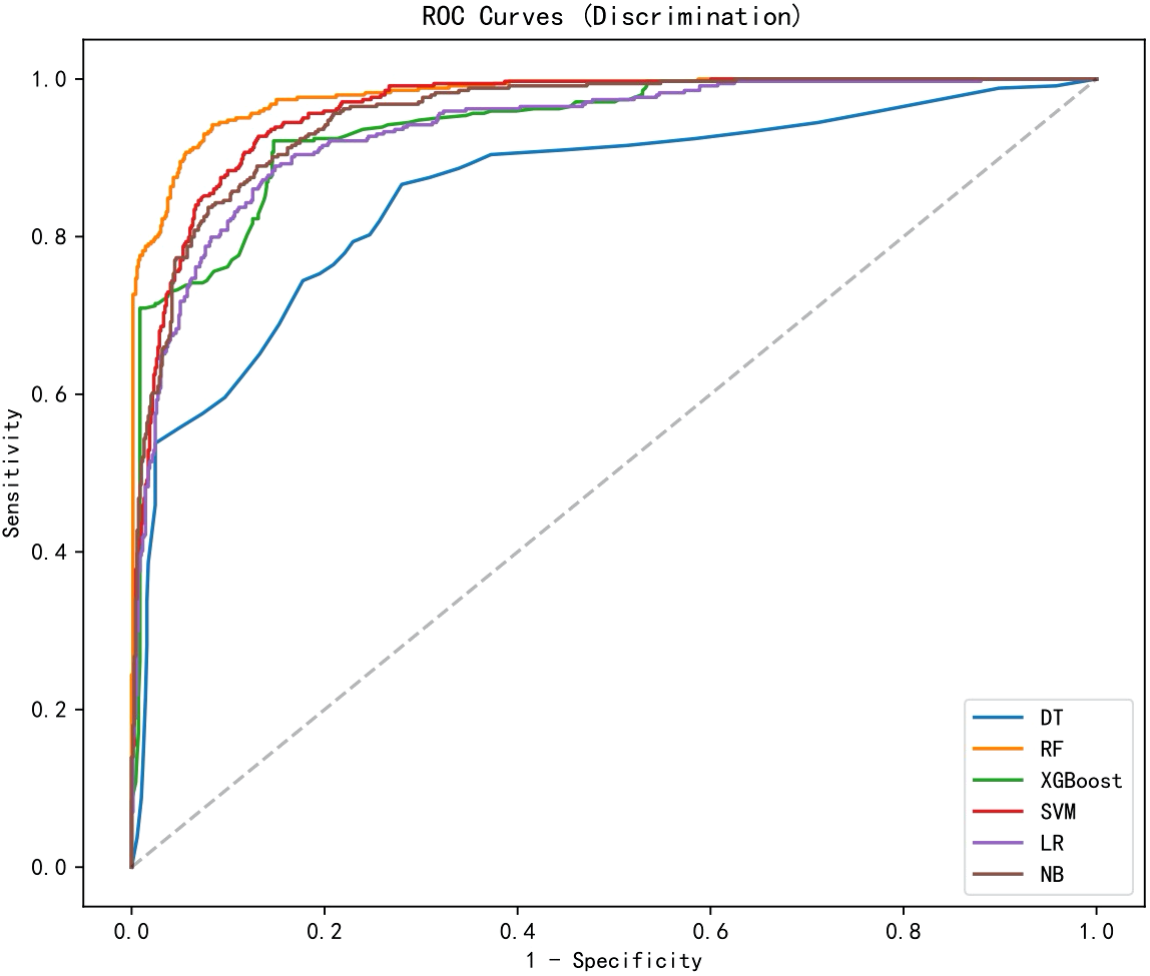
ROC curves of six machine learning models in internal validation (10−fold cross−validation).

To obtain unbiased estimates of model performance while preventing data leakage, we applied the nested 10-fold cross-validation (CV) procedure detailed in the Methods section. In this procedure, feature selection (LASSO with inner CV) and hyperparameter tuning (second-level inner CV) were performed within each outer training fold, and the resulting model was evaluated on the held-out outer validation fold. The aggregated predictions from all 10 outer validation folds were used to compute the metrics below. As shown in **Figure 4**, the Random Forest (RF) model achieved the best discrimination among the six algorithms, with a nested 10−fold cross−validation AUC of 0.979 (95% CI: 0.972--0.986). As shown in **Table 4**, In terms of sensitivity, Although the RF model (0.776) does not perform as well as SVM model (0.852) and NB model (0.858), the RF model also yielded the highest accuracy (0.918), F1 score (0.863), and specificity (0.988). The RF model demonstrated excellent calibration: intercept –0.004 (very close to 0), slope 0.998 (near 1), Brier score 0.074, and HL test p = 0.643 (well above 0.05). The eight predictors were consistently selected across the outer CV folds; the final model presented in the online calculator was refitted on the entire development cohort using these predictors and the optimal hyperparameters identified by the nested CV procedure. This refitting does not affect the unbiased performance estimates reported here.

**Table 4:** Performance comparison of six models in internal validation using nested 10−fold cross−validation (*n* = 1037).

Model	AUC (95% CI)	Accuracy	Sensitivity	Specificity	F1 Score	Brier	HL-P	Slope	Intercept
DT	0.856 (0.829~0.880)	0.830	0.538	0.976	0.678	0.124	0.019	0.926	-0.024
RF	0.979 (0.972~0.986)	0.918	0.776	0.988	0.863	0.074	0.643	0.998	-0.004
XGBoost	0.939 (0.926~0.953)	0.883	0.666	0.991	0.791	0.132	0.000	3.933	1.572
SVM	0.960 (0.949~0.969)	0.901	0.852	0.925	0.850	0.089	0.000	3.926	1.063
LR	0.935 (0.919~0.949)	0.877	0.761	0.934	0.804	0.093	0.120	1.201	0.204
NB	0.953 (0.942~0.964)	0.879	0.858	0.889	0.824	0.084	0.000	0.736	-0.375

**Figure 5:**
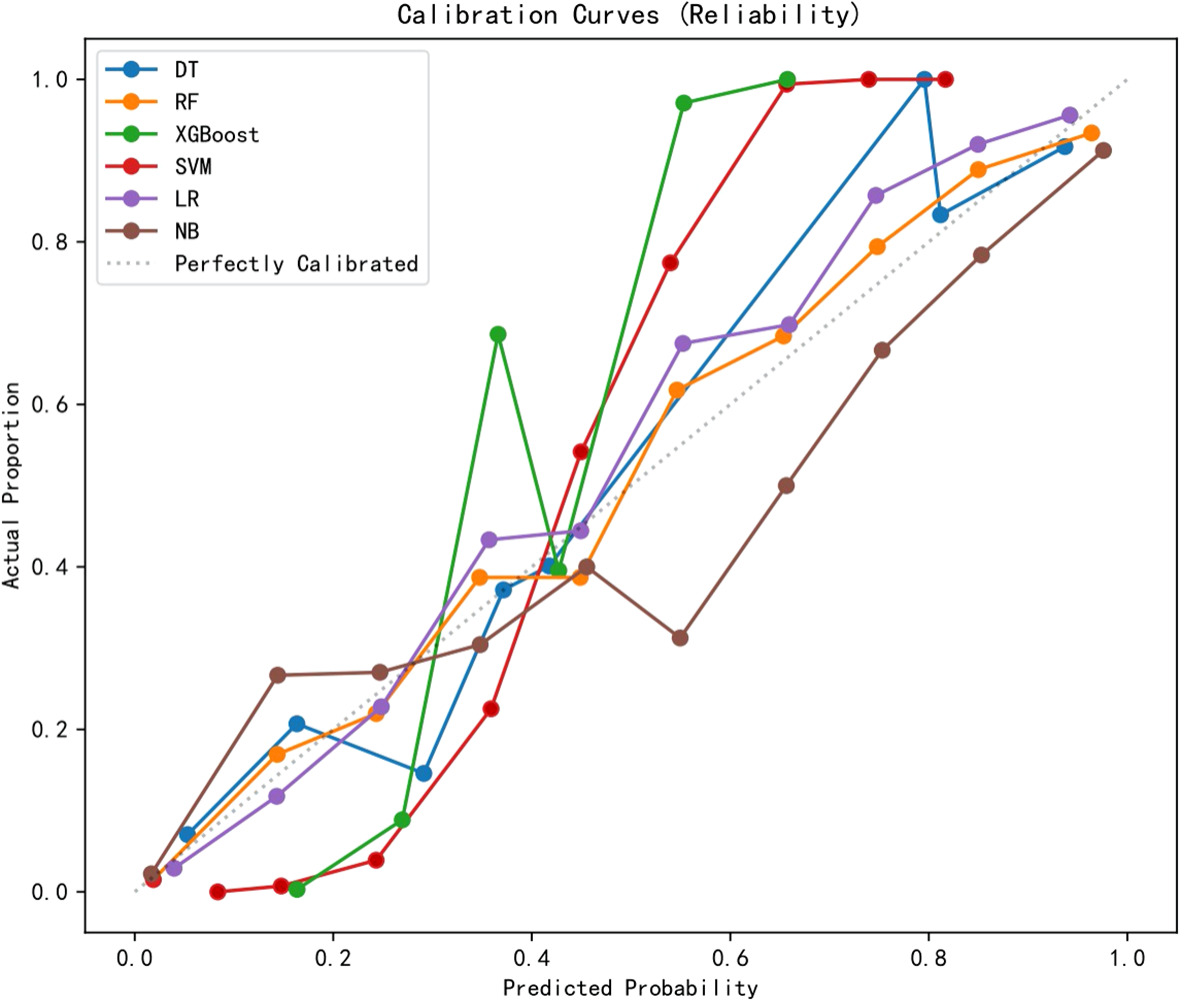
Calibration curves of six machine learning models in internal validation (10−fold cross−validation).

Besides, as show in **Figure 5**, the calibration curve of the Random Forest model closely followed the ideal 45° line, indicating that the predicted probabilities of DFI abnormality were in good agreement with the observed event rates. The Hosmer−Lemeshow test yielded a p−value of 0.643, further supporting no lack of fit.

**Figure 6:**
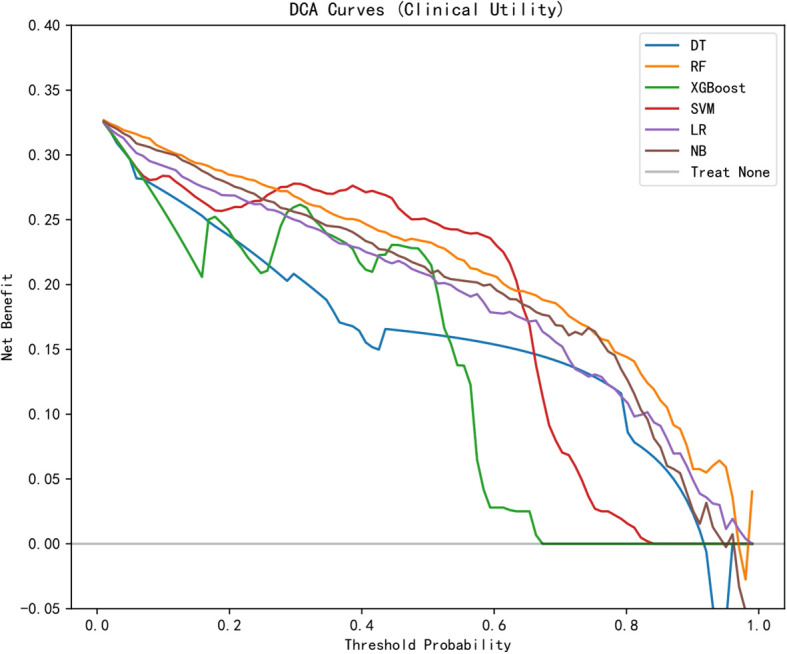
DCA curves of six machine learning models in internal validation (10−fold cross−validation).

In addition, DCA was performed for all six models under the clinical scenario of offering a confirmatory sperm DFI test to patients whose predicted probability of DFI abnormality exceeded a given threshold probability. As shown in **Figure 6**, all six models yielded standardized net benefit above the “treat none” line across a range of thresholds. The RF model demonstrated the most favorable DCA profile among the six algorithms, with a positive standardized net benefit that exceeded both the “treat all” and “treat none” strategies across a clinically plausible threshold range of approximately 10–60%. In this range, the RF curve was consistently above the other models, and its descent was smoother (i.e., net benefit decreased more gradually at higher thresholds), indicating better overall clinical utility. For thresholds below 10%, the “treat all” strategy provided higher net benefit, while for thresholds above 70%, standardized net benefit approached zero. Importantly, these findings apply only to the decision of whether to order a confirmatory DFI test; they do not directly inform other clinical decisions such as ART strategy (e.g., ICSI vs. conventional IVF) or therapeutic interventions. The exact threshold and magnitude of net benefit depend on local costs, availability of DFI testing, and patient preferences, none of which were directly assessed. Therefore, the DCA results should be considered exploratory.

### 3.4 External validation of the model

**Figure 7:**
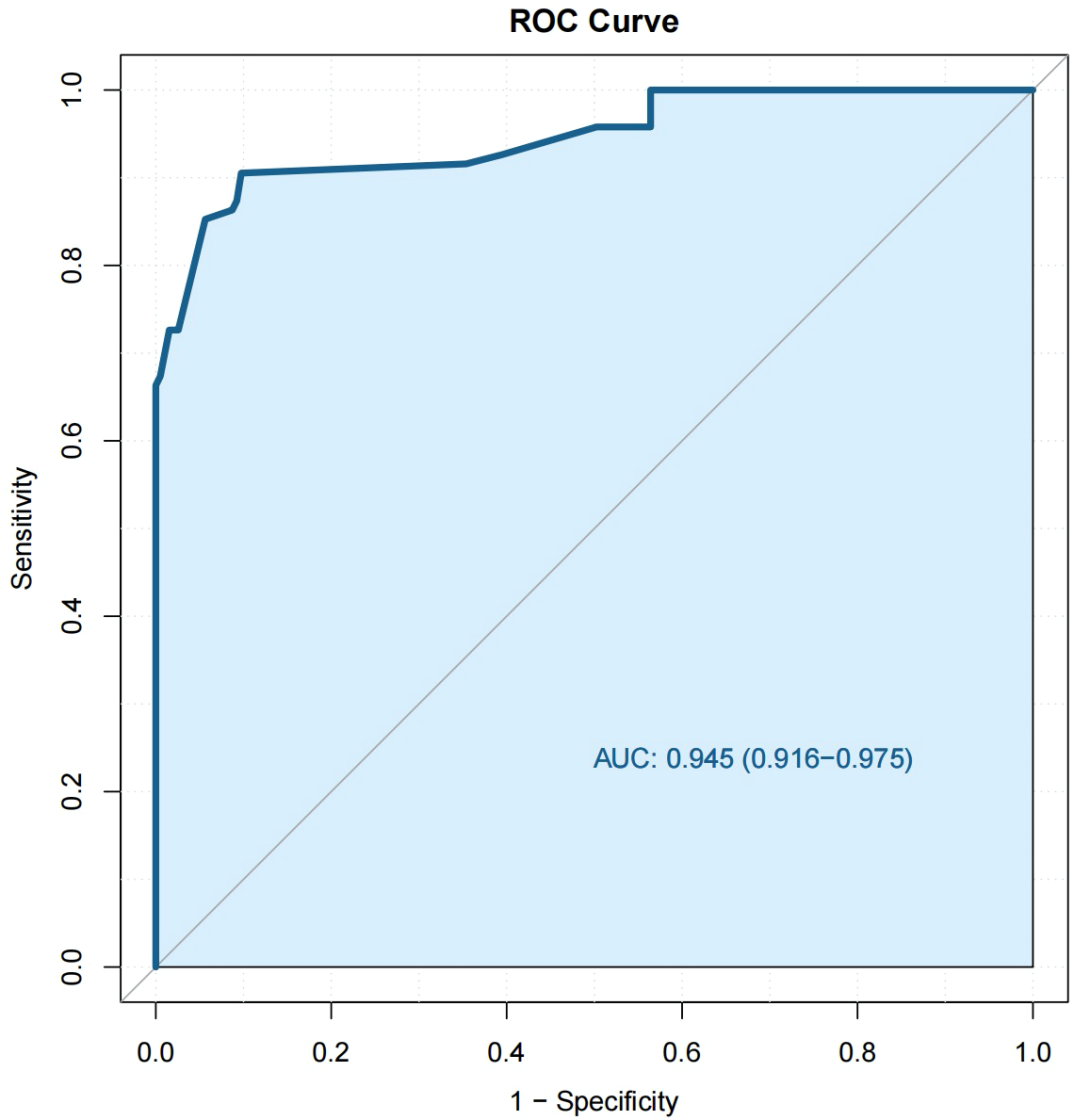
ROC curve of the external validation set for the RF model.

The final RF model (trained on the full development cohort, n = 1037) was applied directly to the independent external validation cohort (Center 2, n = 290) without any modification. As show in **Figure 7**, the RF model achieved an AUC of 0.945 (95% CI: 0.916–0.975), accuracy 0.897, sensitivity 0.863, specificity 0.913, and Brier score 0.093.

**Figure 8:**
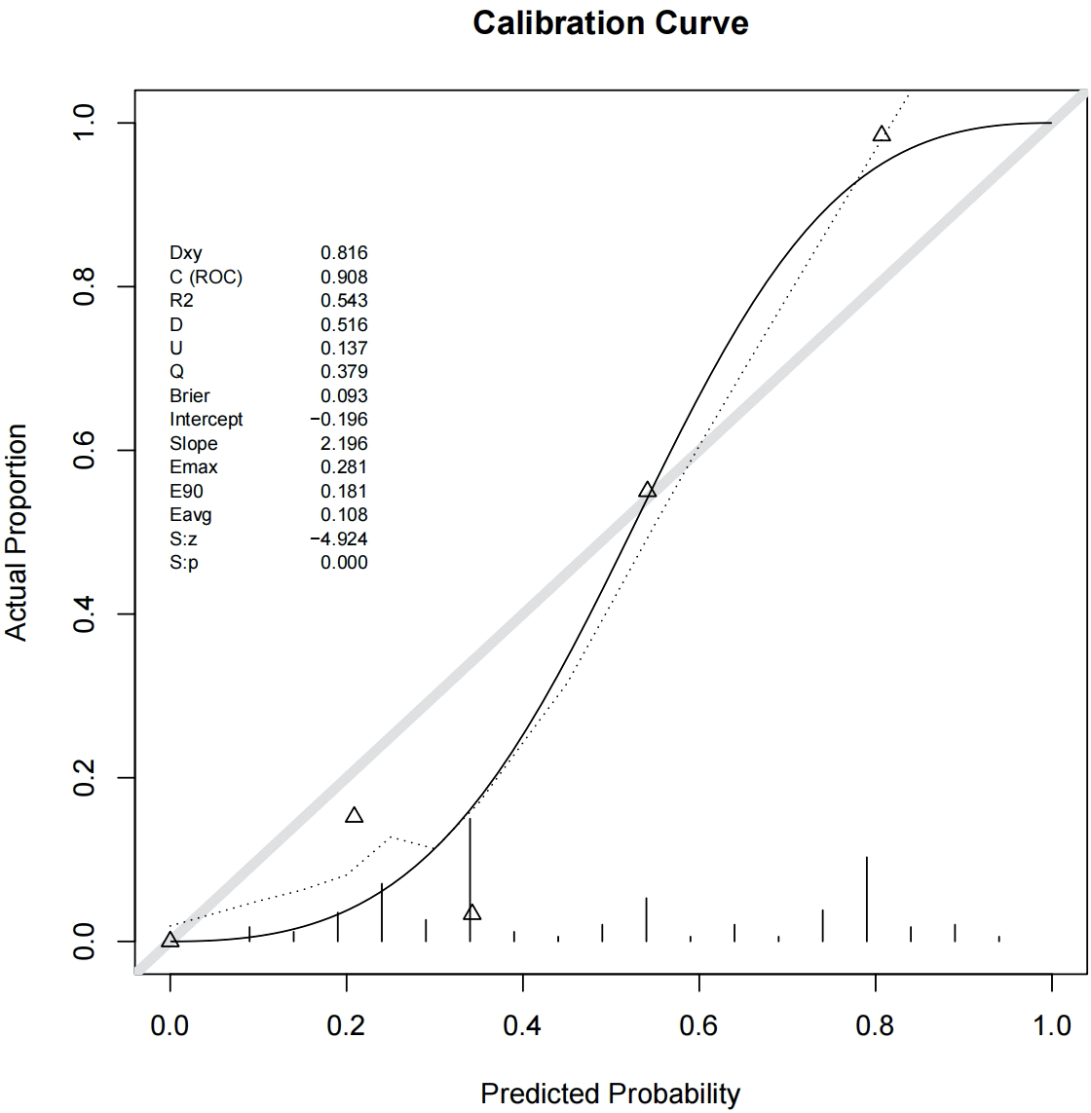
Calibration curve of the RF model’s external validation set.

However, as shown in **Figure 8**, the RF model showed less favorable calibration on the external validation set: the calibration intercept was -0.196 and the calibration slope was 2.196. According to standard calibration methodology, a negative intercept indicates that the model systematically overestimates the overall risk of DFI abnormality in the external population (i.e., predicted risks are, on average, higher than observed event rates). A calibration slope greater than 1 indicates that the predicted probabilities are insufficiently dispersed (too moderate). As a result, for low-risk patients, the model overestimates the risk (predicted probabilities are higher than observed), while for high-risk patients, the model underestimates the risk (predicted probabilities are lower than observed). This pattern of miscalibration is commonly observed when a model developed in one center is applied to a different population, and it underscores the need for future recalibration (e.g., intercept adjustment and slope recalibration) before clinical deployment.

**Figure 9:**
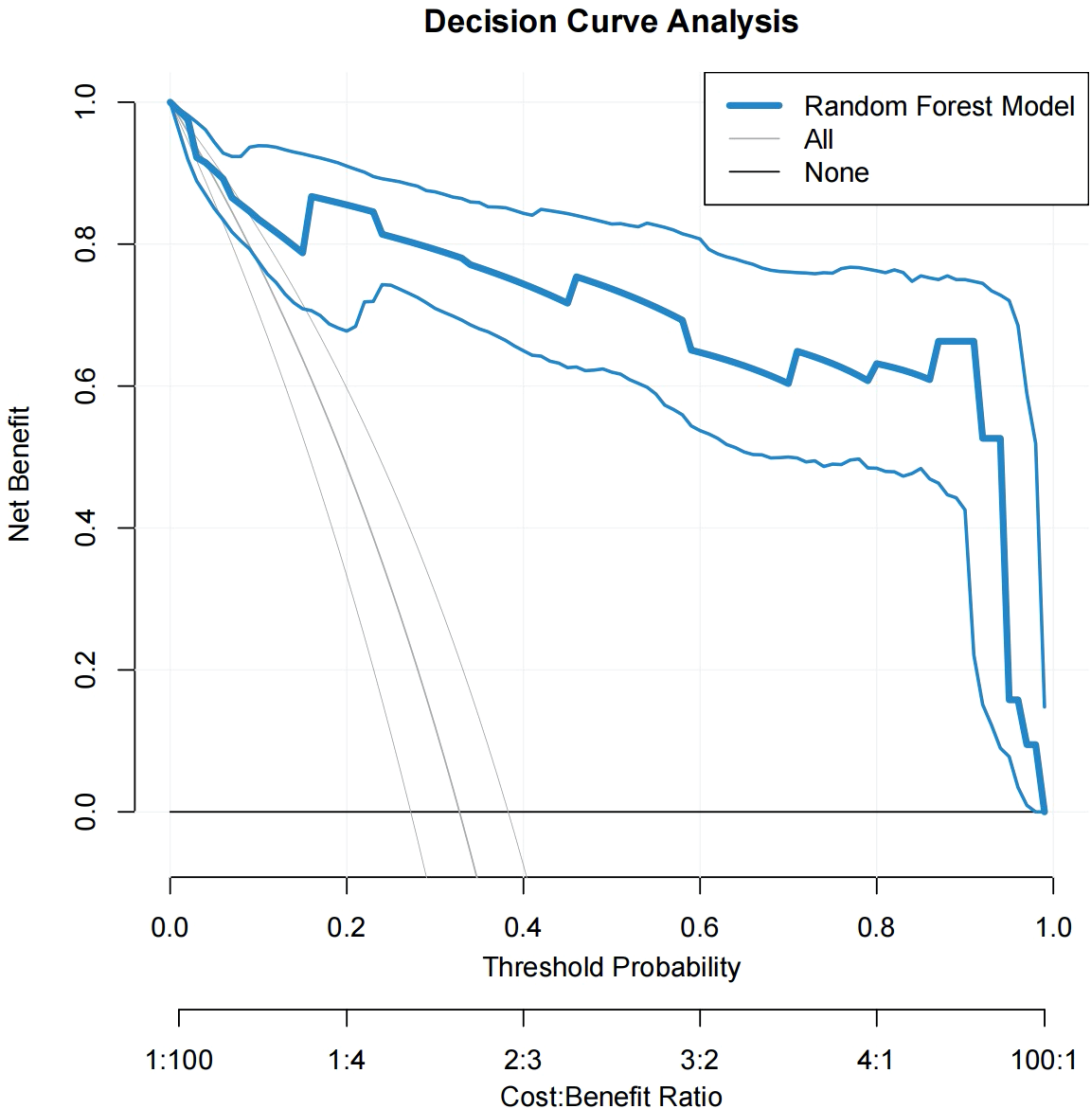
Decision curve analysis (DCA) curve for the external validation set of the RF model.

As shown in **Figure 9**, under the same clinical decision scenario (offering a confirmatory DFI test based on predicted risk), the external validation DCA for the RF model showed that the standardized net benefit curve remained above the “treat all” and “treat none” lines for threshold probabilities approximately between 15% and 75%, consistent with the internal validation pattern. The curve is smooth without irregular fluctuations. However, given the significant miscalibration observed (calibration intercept –0.196, slope 2.196), these DCA results should be interpreted with caution: a miscalibrated model can produce optimistic or misleading net benefit estimates, particularly at extreme thresholds. Therefore, this DCA analysis does not provide evidence of clinical utility in the model’s current form. Recalibration (e.g., intercept adjustment and slope correction) followed by prospective validation is required before any clinical deployment.

### 3.5 Visualization of models

**Figure 10:**
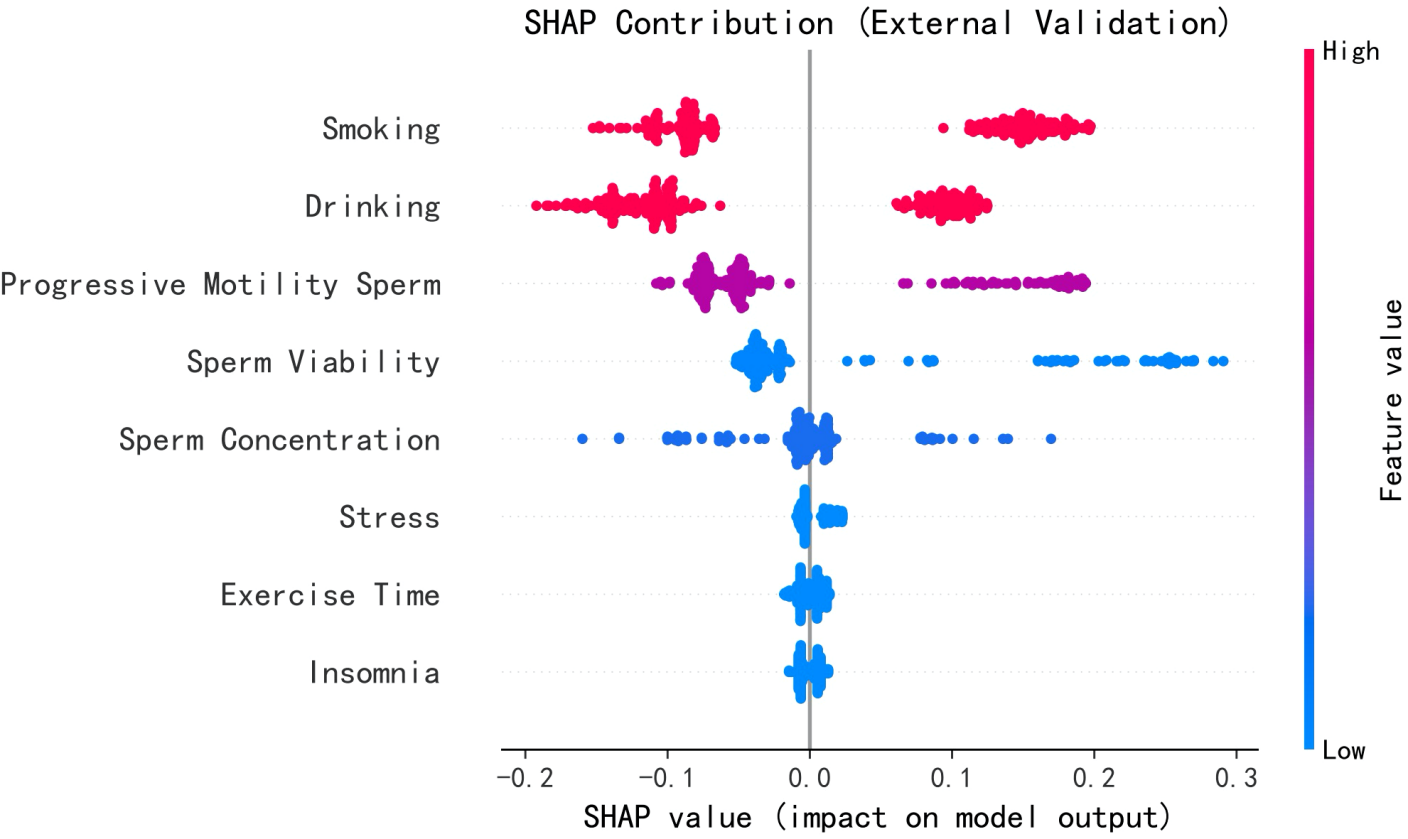
SHAP analysis of the RF model for predicting DFI quality in infertile male patients.

**Figure 11:**
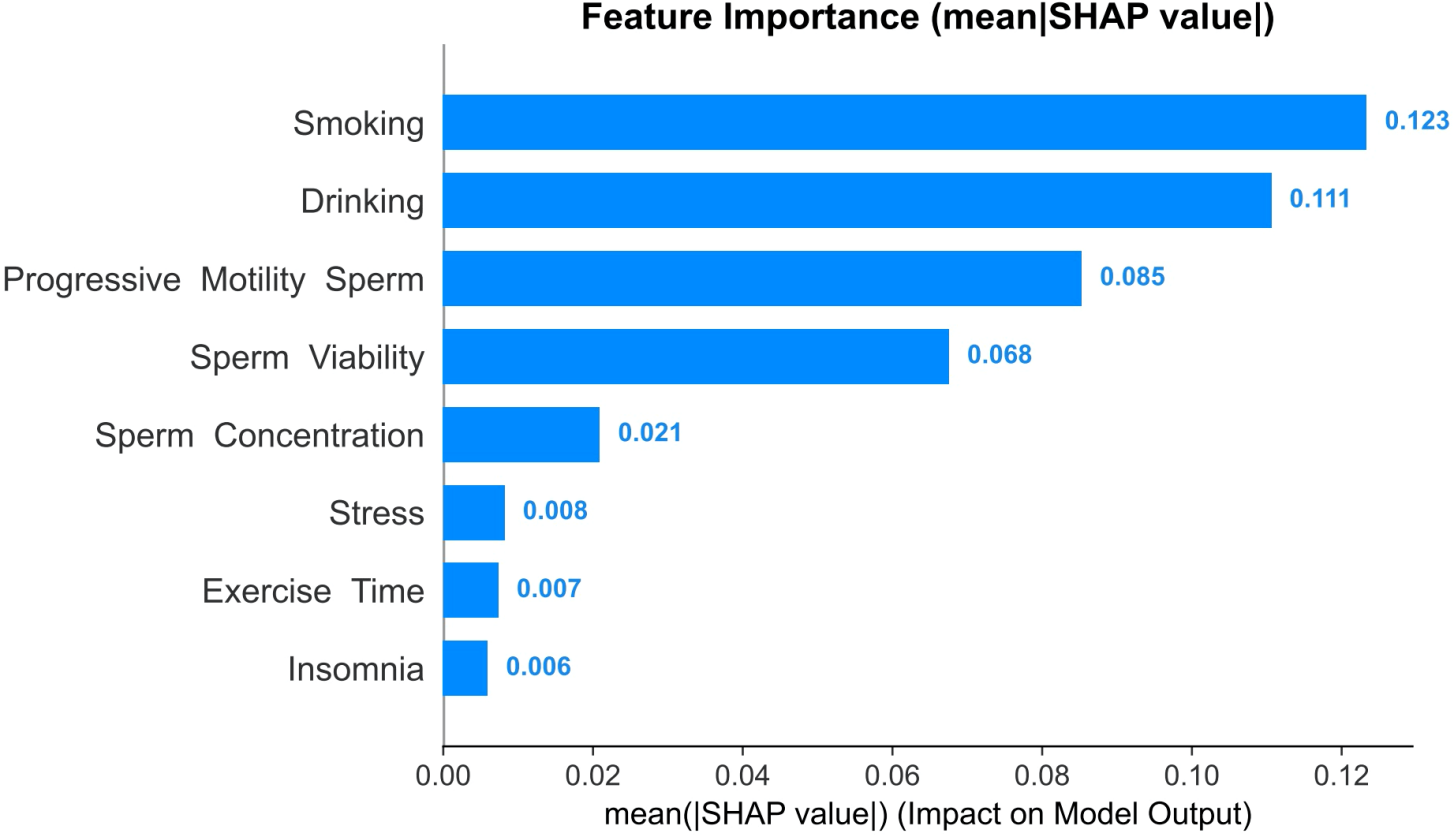
RF model variable importance ranking.

SHAP diagram is used to visualize the impact of predicted variables on model results, depicting the contributions of multiple factors to the predicted results, generate a histogram based on the importance ranking of features, as shown in **Figures 10**, **11**. Using these eight key indicators, we developed a web-based calculator to facilitate individualized risk assessment of DFI quality in infertile male patients, improving accessibility and convenience ((https://4sjajo-0-0.shinyapps.io/dfi-prediction-v4/). This calculator is provided for research purposes only; it should not be used for clinical decision−making until the model has been recalibrated and prospectively validated in the intended−use population.

## 4 Discussion

Male infertility is a global public health issue that has a significant impact on reproductive health and family well-being. DFI is an important indicator for evaluating male fertility, especially for couples whose semen routine analysis (such as sperm count and motility) is normal but who have not yet successfully conceived. Sperm DNA fragment testing can help identify whether sperm DNA damage is a potential cause of infertility (14). In addition to intrinsic factors such as oxidative stress and abnormal apoptosis (15–17), external factors such as unhealthy lifestyle habits, environmental pollution, reproductive system infections, and certain iatrogenic factors (such as chemotherapy or radiation therapy) can also cause damage to sperm DNA fragments (18, 19). Although DFI testing holds significant diagnostic value, its clinical application remains limited due to stringent equipment and environmental requirements, high costs, and the combined consideration of medical resource allocation and patient financial burden. Therefore, developing a clinically practical predictive model with robust performance could not only optimize the diagnostic workflow for male infertility but also provide scientific evidence for precision medicine and personalized interventions. This study employed Lasso regression to identify eight variables influencing DFI quality in infertile males: stress, insomnia, alcohol consumption, and smoking demonstrated inhibitory effects on DFI quality, whereas progressive motile sperm rate, sperm concentration, sperm survival rate, and daily exercise duration showed positive associations with improved DFI quality.

Morphologically normal sperm are more likely to possess intact genetic material. Chua et al. (20) categorized 2,564 men undergoing semen analysis at a medical center in Perth, Australia, into five groups based on DFI levels (<5%, 5.1%-10%, 10.1%-15%, 15.1%-29.9%, and ≥30%), and found that DFI was negatively correlated with both normal sperm morphology rate and progressive motility. In 2024, Zhang et al. (15) demonstrated that the high DFI group exhibited increased abnormal sperm morphology rates and reduced total progressive motile sperm counts, which were associated with elevated reactive oxygen species (ROS) generation during sperm DNA fragmentation. ROS can attack both nuclear and mitochondrial DNA, leading to compromised sperm quality. Reactive oxygen species in seminal plasma are primarily generated by leukocytes and defective sperm present in the ejaculate (21). In healthy males, there exists a dynamic equilibrium between ROS production and antioxidant defense capacity. When this balance is disrupted, excessive ROS accumulation occurs. The abundant polyunsaturated fatty acids in sperm membranes become susceptible to lipid peroxidation under oxidative stress, while oxidative damage simultaneously causes DNA strand breaks and impairs sperm DNA integrity. Additionally, semen samples with low sperm motility or survival rates exhibit higher proportions of apoptotic or defective sperm. These dysfunctional sperm cells generate ROS, reduce mitochondrial membrane potential, decrease ATP production, and consequently elevate the sperm DNA fragmentation index.

Unhealthy lifestyle factors such as smoking, alcohol consumption, insomnia, stress, and physical inactivity primarily contribute to decreased DFI quality by elevating oxidative stress levels and generating free radicals that damage sperm cell membranes and nuclear DNA (6). Although the mechanisms through which smoking induces sperm DNA damage remain unclear, toxic substances in tobacco smoke—including tar, nicotine, and carbon monoxide—exhibit reproductive toxicity. These compounds impair hypothalamic-pituitary axis function, reduce sex hormone secretion, decrease cellular checkpoint kinase 1 (Chk1) expression in males, and consequently trigger sperm DNA damage and apoptosis (15). Chronic excessive alcohol intake causes testicular toxicity and degeneration, leading to abnormalities in androgen and gonadotropin levels, which negatively affect spermatogenesis, differentiation, and development processes (21), thereby compromising DFI quality. Gill et al. (16) demonstrated that men spending ≥50% of working hours sedentary exhibited significantly higher sperm DFI compared to those with <50% sedentary time, indicating that prolonged sitting induces local circulatory disturbances in the perineal region and metabolic waste accumulation, resulting in sperm DNA fragmentation. Another study revealed that long-term (12-week) aerobic exercise enhances transient receptor potential ankyrin 1 (TRPA1) expression in epididymal epithelium of diet-induced obese rats. TRPA1 activation promotes Ca²^+^ influx and transepithelial secretion of Cl^-^ and HCO_3_^-^, creating a microenvironment essential for sperm maturation and improving sperm quality (22). Sleep deprivation activates the hypothalamic-pituitary-gonadal (HPG) axis (23) and increases serotonin secretion (24), which suppresses testosterone production and induces apoptosis in Leydig cells. Additionally, chronic stress disrupts endocrine homeostasis by causing hormonal imbalances and neurotransmitter metabolism disorders involving monoamines and peptides, subsequently dysregulating secretory functions of both the hypothalamic-pituitary-adrenal (HPA) axis and HPG axis (25), ultimately impairing spermatogenesis.

Machine learning is a scientific approach that focuses on how computer systems can learn and improve from given data based on complex algorithms to make and optimize predictions, which traditional statistical methods struggle to achieve (26). The main steps of machine learning involve introducing an algorithm, extracting input data, making accurate predictions through computer analysis, identifying relevant features from the data, and relearning from previous experiences (26). In recent years, related studies have utilized machine learning algorithms to predict sperm quality. For instance, KURODA et al. (27) trained a deep learning model based on convolutional neural networks using over 1,000 acridine orange-stained sperm images and corresponding DFI values measured by experimental techniques. The model’s measurements showed no systematic or proportional differences compared to manual measurements. Additionally, in terms of DFI prediction, existing research has employed trained machine learning models to detect DFI (28). These models utilized data from sperm images and demonstrated that sperm morphology is associated with sperm DNA integrity, achieving an accuracy of 0.827 on the test set. This study confirms that machine learning can effectively integrate nonspecific indicators such as semen quality and lifestyle to predict DFI quality and enhance diagnostic performance to some extent. Furthermore, this study adopted 10-fold cross-validation and Bootstrap resampling methods to effectively control the risk of overfitting and ensure the model’s generalization ability. Among the six compared machine learning algorithms, the RF model demonstrated the best performance, with a 10−fold cross−validation AUC of 0.979 in the development cohort and an external validation AUC of 0.945 (F1 score = 0.863 for internal validation). The reason lies in the deep integration of methodology and clinical thinking in the constructed predictive model. Firstly, Lasso regression was used for variable selection according to the lambda.1se criterion, ensuring the inclusion of the strongest predictive variable combinations. Secondly, RF, as the optimal model in this study, efficiently handles datasets with multivariate and high-dimensional characteristics through ensemble learning, making it suitable for the comprehensive data in this study. RF exhibited highly consistent performance between the training and test sets, and while the external validation AUC slightly decreased, it still maintained good predictive capability, indicating strong robustness against data noise. Moreover, the RF algorithm offers advantages such as ease of use, fast training, and prevention of overfitting, reducing the need for extensive hyperparameter tuning. In addition, for further application, we transform complex prediction algorithms into intuitive and easy-to-use clinical tools through network-based calculators. This tool has the potential to assist clinical urologists in risk stratification, developing personalized treatment plans, optimizing resource allocation, and improving communication with patients and their families. The DCA results presented in this study are strictly limited to the hypothetical decision of whether to order a confirmatory sperm DFI test based on the model’s predicted risk. We explicitly caution against interpreting these results as evidence that the model can guide treatment selection (e.g., antioxidant therapy, lifestyle counseling) or ART strategy (e.g., ICSI versus conventional IVF). Furthermore, because the model exhibits significant miscalibration on external validation (calibration slope 2.196, intercept -0.196), the DCA estimates may be optimistic or misleading. Therefore, in its current form, the model should not be used for any clinical decision-making, including DFI test triage. Recalibration and prospective validation are necessary prerequisites before any future evaluation of clinical utility.

Several limitations should be considered. First, despite the rigorous internal and external validation, both centers are located in Shanghai, which may limit generalizability to other populations. Second, the model was developed using a retrospective design; prospective validation is warranted. Third, we did not perform imputation for missing data, although the missing rate was very low and sensitivity analysis showed robustness. Fourth, external calibration of the RF model showed a calibration intercept of -0.196 and a slope of 2.196. A negative intercept indicates that the model systematically overestimates the overall risk of DFI abnormality in the external validation population. A calibration slope greater than 1 indicates that the predicted probabilities are insufficiently dispersed (too moderate). Consequently, the model overestimates risk for low-risk patients and underestimates risk for high-risk patients. This pattern of miscalibration is not unexpected when a model developed in one center is applied to a different population with different case mix or data collection procedures. Therefore, before this model can be recommended for clinical deployment, it should undergo recalibration (e.g., intercept adjustment and slope recalibration) followed by prospective validation in the intended-use population. Fifth, and most importantly, this model predicts DFI abnormality, which is an intermediate endpoint. While DFI has been consistently associated with ART outcomes, it does not directly measure reproductive success such as live birth. Therefore, the clinical utility of our model—whether it can guide interventions to improve patient−important outcomes—remains to be proven in future prospective studies. We caution against overinterpreting the current results as direct evidence of clinical benefit. The model’s current miscalibration and the lack of prospective evidence linking model−guided decisions to improved reproductive outcomes (e.g., live birth) mean that the model and its associated online calculator should be used for research purposes only. Any claim of clinical utility or readiness for practice would be premature at this stage.

## 5 Conclusion

This study successfully developed a predictive model for DFI abnormality in infertile men using the Random Forest algorithm. The model demonstrated good discrimination in internal validation (nested 10-fold cross-validation AUC = 0.979) and fair discrimination in external validation (AUC = 0.945). However, external calibration revealed systematic miscalibration (calibration slope 2.196, intercept –0.196), indicating overall overestimation of risk and insufficient dispersion of predicted probabilities. Therefore, in its current form, the model and its associated online calculator should be considered investigational and should not be used for standalone clinical decision-making. The model predicts an intermediate endpoint (DFI abnormality), not patient-important reproductive outcomes such as live birth or clinical pregnancy. Future work must focus on recalibration (e.g., intercept adjustment and slope recalibration) followed by prospective validation in larger, multi-center populations, with direct assessment of impact on patient-important outcomes. Only after such evidence is available can the model be considered for clinical deployment. Nonetheless, this study provides a methodological framework that, after further refinement and validation, may eventually contribute to precision medicine in male infertility.

## Data Availability

The datasets used in this study are not publicly available due to privacy and ethical restrictions and cannot be shared. Requests to access the datasets should be directed to 122710487@qq.com.
